# Editorial: The role of affect regulation in bipolar disorders

**DOI:** 10.3389/fpsyt.2025.1659734

**Published:** 2025-08-11

**Authors:** Magnus Johan Engen, Trine Vik Lagerberg, Margrethe Collier Høegh

**Affiliations:** ^1^ Nydalen District Psychiatric Center, Division of Mental Health and Addiction, Oslo University Hospital, Oslo, Norway; ^2^ Department of Research and Development, Division of Mental Health and Addiction, Oslo University Hospital, Oslo, Norway; ^3^ Department of Psychology, Faculty of Social Sciences, University of Oslo, Oslo, Norway; ^4^ Section for Psychosis Research, Division of Mental Health and Addiction, Oslo University Hospital, Oslo, Norway

**Keywords:** bipolar disorders (BD), affect regulation, clinical interventions, biological mechanisms, perspectives, research targets, treatment models

Bipolar disorders (BD) are a group of complex mental illnesses with a lifetime prevalence of 2.4% (1), characterized by illness episodes with extreme alterations in mood and activity that typically emerge in early adulthood. Despite early detection and psychopharmacological treatment to manage episodes, many individuals respond only partially to treatment and continue to experience affective symptoms and impaired functioning. Difficulties with affect regulation are increasingly being highlighted as a pathway to understanding the persistent gaps in treatment outcomes (2–5). This change in focus is welcome and follows an important and emerging trend where affective dysregulation is seen as clinically significant transdiagnostic features, with important implications for both understanding and treatment across different mental disorders.

In BD, affective dysregulation including affective lability, defined as rapid, excessive and unpredictable fluctuations between different affective states (6), is observed prior to illness onset, during both depressed and elevated mood episodes, as well as during euthymic periods throughout the course of illness (7–12). It is linked to childhood trauma—which is prevalent in the BD population—and associated with anxiety, suicidality, impaired functioning and substance use (13–16). It is important to recognize the role of affective dysregulation as a key contributing factor to complex clinical presentations, particularly in cases where state-of-the-art pharmacological treatments do not adequately alleviate symptoms or improve quality of life. Viewing core aspects of BD through this transdiagnostic lens also opens new opportunities to draw on advances in treatment and conceptual frameworks from other areas in mental health care. In this Research Topic, we proudly present a collection of papers that serve to advance our understanding of the role of affect regulation in BD.

In this Research Topic, a perspective article by Azevedo et al. provides an overview of how the European Network for Bipolar Emotion Regulation views the current evidence on affective dysregulation in BD and outlines specific steps to help bridge the gap between research and clinical practice. Importantly, they note that treatments such as Dialectical Behavior Therapy have already demonstrated effectiveness in improving affect regulation and supporting personal recovery in BD. To further support the development of interventions targeting affect regulation, the authors advocate for a greater focus on personal recovery and quality of life and emphasize the value of increasing user involvement in research design.

In line with this, Engen et al. present a feasibility study using both qualitative interviews and quantitative outcome data to evaluate the transdiagnostic treatment model the Unified Protocol (UP), delivered in a group setting. This pilot included patients with BD early in the course of treatment and found significant reductions in affective lability, along with improvements in well-being and functioning. The study concludes by outlining specific refinements to the treatment format, informed by insights from the participants.

An important study by Visalli et al. shows how dysregulated affect can impact outcomes through poorer self-care and reduced adherence to treatment. Their results indicate that hyperthymic temperament—as opposed to depressive or cyclothymic temperaments—was associated with better treatment adherence. Tokumitsu et al. further elaborate on the complex relationship between clinical features and illness course in a large Japanese outpatient sample (N = 2,392). In this study, alcohol dependence was found to be associated with BD I, manic episodes, comorbid psychiatric disorders, and suicidal ideation, especially among women. These studies highlight the relationship between dysregulated affect and maladaptive coping strategies, with important implications for intervention- and treatment planning.

The biological underpinnings of affective dysregulation are thoroughly reviewed in a paper by Durdurak et al. This narrative review describes how affective dysregulation in BD is linked to factors such as genetics, brain structure, neuroinflammation, circadian rhythm disturbances, and abnormalities in neuroplasticity. This comprehensive overview invites readers to consider the broad and interconnected biological mechanisms underlying affective dysregulation, while the paper by Wang et al. specifically investigates how the immune system biomarker Interleukin-17 (IL-17) is implicated in affective disturbances in BD.

Together, these contributions illustrate the diverse and multifaceted nature of affect regulation in BD. From biological mechanisms and clinical interventions to psychosocial influences and emerging treatment models, the papers in this Research Topic collectively underscore the importance of targeting affect regulation as a core component of future frameworks for understanding and treating BD.
